# Targeting the PI3K/Akt Cell Survival Pathway to Induce Cell Death of HIV-1 Infected Macrophages with Alkylphospholipid Compounds

**DOI:** 10.1371/journal.pone.0013121

**Published:** 2010-09-30

**Authors:** Amanda Lucas, Yuri Kim, Omayra Rivera-Pabon, Sunju Chae, Dong-Hyun Kim, Baek Kim

**Affiliations:** 1 Department of Microbiology and Immunology, University of Rochester Medical Center, Rochester, New York, United States of America; 2 Department of Pharmacy, College of Pharmacy, Kyung-Hee University, Seoul, South Korea; University of Cambridge, United Kingdom

## Abstract

**Background:**

HIV-1 infected macrophages and microglia are long-lived viral reservoirs persistently producing viral progenies. HIV-1 infection extends the life span of macrophages by promoting the stress-induced activation of the PI3K/Akt cell survival pathway. Importantly, various cancers also display the PI3K/Akt activation for long-term cell survival and outgrowth, and Akt inhibitors have been extensively searched as anti-cancer agents. This led us to investigate whether Akt inhibitors could antagonize long-term survival and cytoprotective phenotype of HIV-1 infected macrophages.

**Principal Findings:**

Here, we examined the effect of one such class of drugs, alkylphospholipids (ALPs), on cell death and Akt pathway signals in human macrophages and a human microglial cell line, CHME5, infected with HIV-1 BaL or transduced with HIV-1 vector, respectively. Our findings revealed that the ALPs, perifosine and edelfosine, specifically induced the death of HIV-1 infected primary human macrophages and CHME5 cells. Furthermore, these two compounds reduced phosphorylation of both Akt and GSK3β, a downstream substrate of Akt, in the transduced CHME5 cells. Additionally, we observed that perifosine effectively reduced viral production in HIV-1 infected primary human macrophages. These observations demonstrate that the ALP compounds tested are able to promote cell death in both HIV-1 infected macrophages and HIV-1 expressing CHME5 cells by inhibiting the action of the PI3K/Akt pathway, ultimately restricting viral production from the infected cells.

**Significance:**

This study suggests that Akt inhibitors, such as ALP compounds, may serve as potential anti-HIV-1 agents specifically targeting long-living HIV-1 macrophages and microglia reservoirs.

## Introduction

Targeting the actions of Human Immunodeficiency Virus Type 1 (HIV-1) proteins is currently a major anti-viral strategy that has led to effective controls of HIV-1 replication and pathogenesis. Unfortunately, this anti-HIV-1 strategy becomes ineffective due to the robust evolution and escape capacity of HIV-1, in which viral populations resistant to the currently available antiviral agents are selected. New anti-HIV-1 strategies which may avoid this viral escape are being extensively researched, and one promising strategy is to target host factors and cellular mechanisms that HIV-1 hijacks for its replication and pathogenesis.

HIV-1 infected macrophages exhibit extended life spans, allowing these cells to become long-lived HIV-1 reservoirs that persistently produce virus [1]. In addition, HIV-1 infected human microglia, resident macrophages of the central nervous system (CNS), isolated from patients displayed enhanced survival compared to uninfected microglia isolated from the same patients [2]. Importantly, it is known that HIV-1 infected macrophages and microglia secrete nitric oxide and various toxic viral proteins, such as gp120 and Tat, establishing cytotoxic extracellular environments near the infected cells [2]. Numerous studies reported that in the brain, these HIV-1 related toxic molecules induce the death of nearby neurons, ultimately leading to HIV-associated neurodegenerative diseases (HAND) in HIV-1 infected patients [3], [4]. However, it is not clearly understood how HIV-1 infected macrophages and microglia are able to live for a long period of time and persistently produce viral progenies while these infected cells are also constantly exposed to the same cytotoxic environments that kill the nearby neurons.

To understand the paradox between the long-lived survival phenotype of HIV-1 infected macrophages and the constant exposure of the cells to the toxic extracellular conditions, we hypothesized that HIV-1 may activate cellular pathways related to cell survival in infected macrophages and microglia. Indeed, we recently reported that HIV-1 infection triggers the activation of the PI3K/Akt cell survival pathway in primary human macrophages and renders these cells resistant to cytotoxic insults [5]. In normal cells without exposure to cellular insults, this pathway remains inactivated by its negative regulator, PTEN [6]. We also demonstrated that the HIV-1 induced cytoprotection is initiated by the expression of an HIV-1 accessory protein, Tat, which lowers the PTEN level in infected macrophages and a human microglia cell line, CHME5 [5]. In the absence of negative regulation of the PI3K/Akt pathway by PTEN, HIV-1 infected macrophages may proactively react to stressful cytotoxic extracellular environments established by the virus-induced chemicals, ultimately elevating their chance of survival.

The PI3K/Akt pathway is also commonly activated in many cancer cells and promotes their survival and outgrowth [7]. Genetic inactivation of the PTEN function, which is oncogenic, activates the PI3K/Akt pathway [8], [9]. Due to the direct mechanistic connection between activation of the PI3K/Akt pathway and cancer, various attempts to screen inhibitors of key enzymes of the pathway such as PI3K and Akt kinases, have been made [10], [11]. Among them, alkylphospholipds (ALPs) have been tested for their anti-cancer effect, and indeed miltefosine was recently found to be effective in the treatment of cutaneous breast cancer metastases [12]. ALPs, also termed antitumor lipids, have been found to display anti-neoplastic effects in various tissue culture and small animal models [11], [13], [14], [15], [16]. Their mechanisms of action involve regulation of multiple cellular events related to cell proliferation, cell death and cell survival [13], [15], [16], [17]. Our recent study reported that miltefosine is able to prevent HIV-1 infected macrophages from activating the PI3K/Akt pathway, and specifically induces death of HIV-1 infected macrophages upon exposure to insults, ultimately terminating viral production [5]. However, the mechanism by which miltefosine counteracts the PI3K/Akt pathway in HIV-1 expressing macrophages remains to be explored.

In this report, we investigated whether the ALP compounds, perifosine and edelfosine, can specifically inhibit the activation of Akt kinase activity in HIV-1 expressing macrophages. Indeed, we demonstrate that perifosine and edelfosine inhibit both the activation of Akt and the kinase activity of Akt in HIV-1 expressing human microglia cell lines, and perifosine effectively induces the death of HIV-1 infected macrophages.

## Methods

### Cells, plasmids, and viruses

Primary human monocyte-derived macrophages were isolated from human buffy coats (New York Blood Center) and differentiated as previously described [5]. M-tropic HIV-1 BaL was provided from Zeptometrix (Buffalo, NY) and VSV-G pseudotyped HIV-1 vector (DHIV-GFP) expressing EGFP and all HIV-1 proteins except Nef and Env were prepared as described [5]. Vector titers were determined with CHME5, a human fetal microglia cell line [18], and the p24 ELISA was performed for each vector or virus preparation following the manufacturer's protocol. pTat101 is a pcDNA3.1+Hygromycin (Invitrogen) derivative expressing a 101 amino acid HIV-1 Tat cDNA derived from the YU-2 M-tropic HIV-1 strain [19]. A CHME5 subline that stably expresses full-length Tat (pTat101) and a control cell line (pCDNA3.1-Hygro) were previously selected [19]. The concentrations of cycloheximide (CHX) and *E. coli* serotype O26:B6 lipopolysaccharide (LPS) (Sigma) used were described in Figure legends.

### Cell survival assays

CHME5 cells were exposed to LPS (50 µg/ml)/CHX (10 µg/ml), and sodium nitroprusside (SNP) (Sigma) was used at 100 µM for treatment of macrophages. Two methods were employed to monitor the dead cell populations. First, the CHME5 sublines were trypsinized and mixed with 0.4% Trypan blue solution (Gibco) at a 1∶1 dilution, and the number of blue (dead) cells was calculated. Second, CHME5 cells and macrophages transduced by the HIV-1 vector expressing GFP were analyzed with ethidium homodimer included in the Live/Dead-Viability/Cytotoxicity kit (Molecular Probes) to stain the dead cells as per the manufacturer's protocol. Images were taken at a 100× magnification at 24 hours post-treatment using a fluorescence microscope (Zeiss). Each assay was performed at least in triplicate. In each assay, three different pictures were taken and >300 transduced green cells (>100 cells per picture) were counted. The number of dead (red) cells per counted transduced (green) cells was used to determine percentage of cell death.

### Assays for phosphorylation and kinase activity of Akt

CHME5 (1×10^6^) cells transduced with pseudotyped HIV-1 vector (∼90% transduction) and CHME5 sublines were treated with LPS (50 µg/ml)/CHX (10 µg/ml) in the presence or absence of ALP compounds for 24 hours. Cells were lysed using ELB lysis buffer before performing the western blot-based Akt kinase activity assay (Cell Signaling) or ELISA-based Akt assay kit (Calbiochem) as per the manufacturers' protocols. The same lysates were used for the western blot for total and phospho-GSK3β as described [5]. Ratios between the band intensities of phosphorylated and non-phosphorylated Akt and GSK3β levels were analyzed using ImageJ software (NIH).

### Virus production assay and live/dead assay with infected macrophages

Primary human macrophages (8×10^4^) were infected with HIV-1 BaL (7×10^4^ pg p24). 24 hours post-infection, cells were washed with Dulbecco's phosphate buffered saline to eliminate the presence of excess virus. After washing, cells were cultured either in media alone, media containing SNP (100 µM), or media supplemented with 20 µM Perifosine (Cayman Chemical). Infected macrophages were cultured for 12 days, and viral production at 12 days post infection was measured by p24 ELISA (Perkin Elmer) according to the manufacturer's protocol. Cells were analyzed for the induction of cell death using the live/dead assay as described above. Merged (red + green + bright field) images are shown at 100× magnification. Green cells represent viable cells while those with red nuclei correspond to dead or dying cells.

## Results

### Effect of ALP compounds on Akt activation and phosphorylation in HIV-1 expressing CHME5 cells

**Figure 1:**
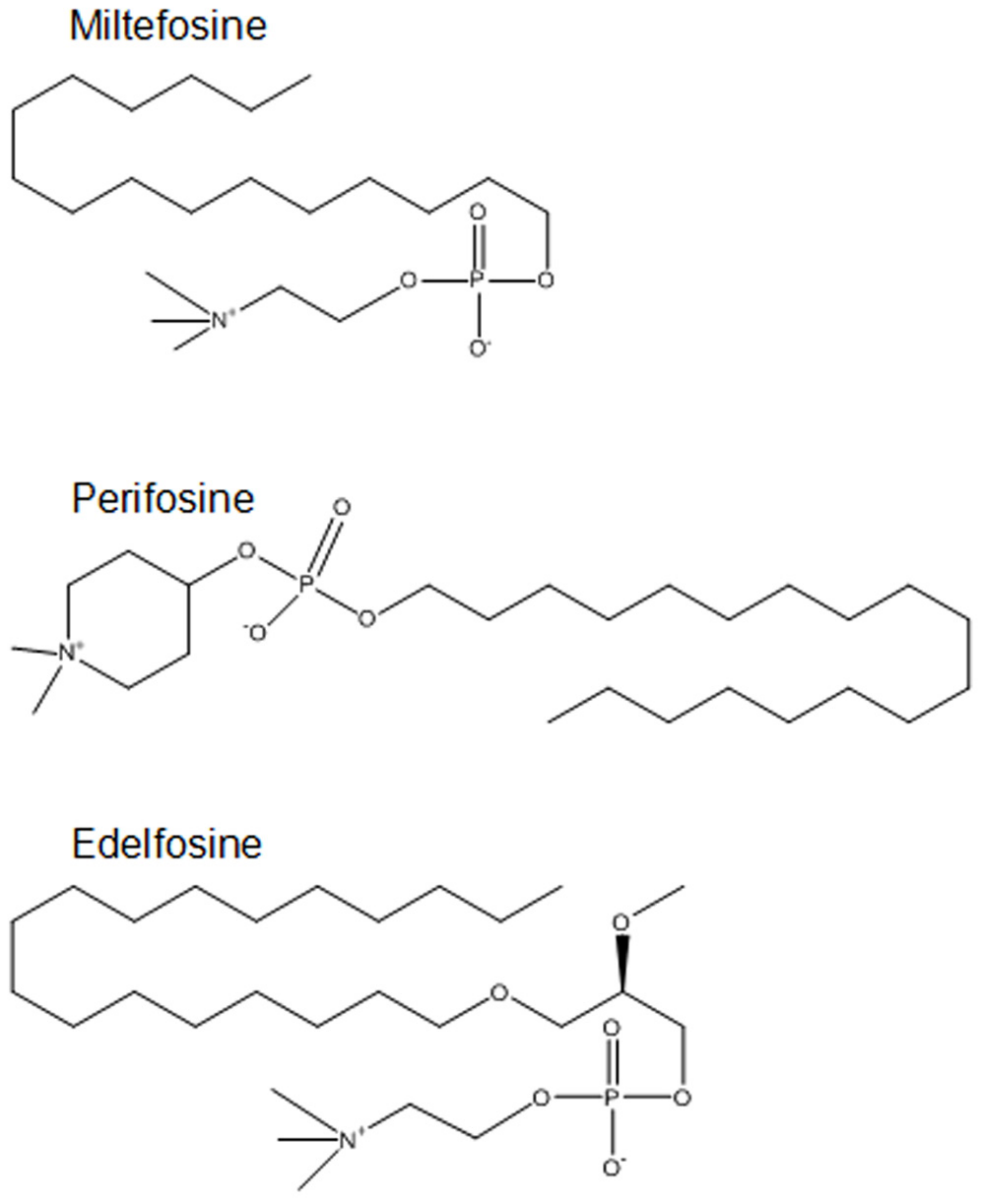
Structures of alkylphospholipid compounds used in this study.

**Figure 2:**
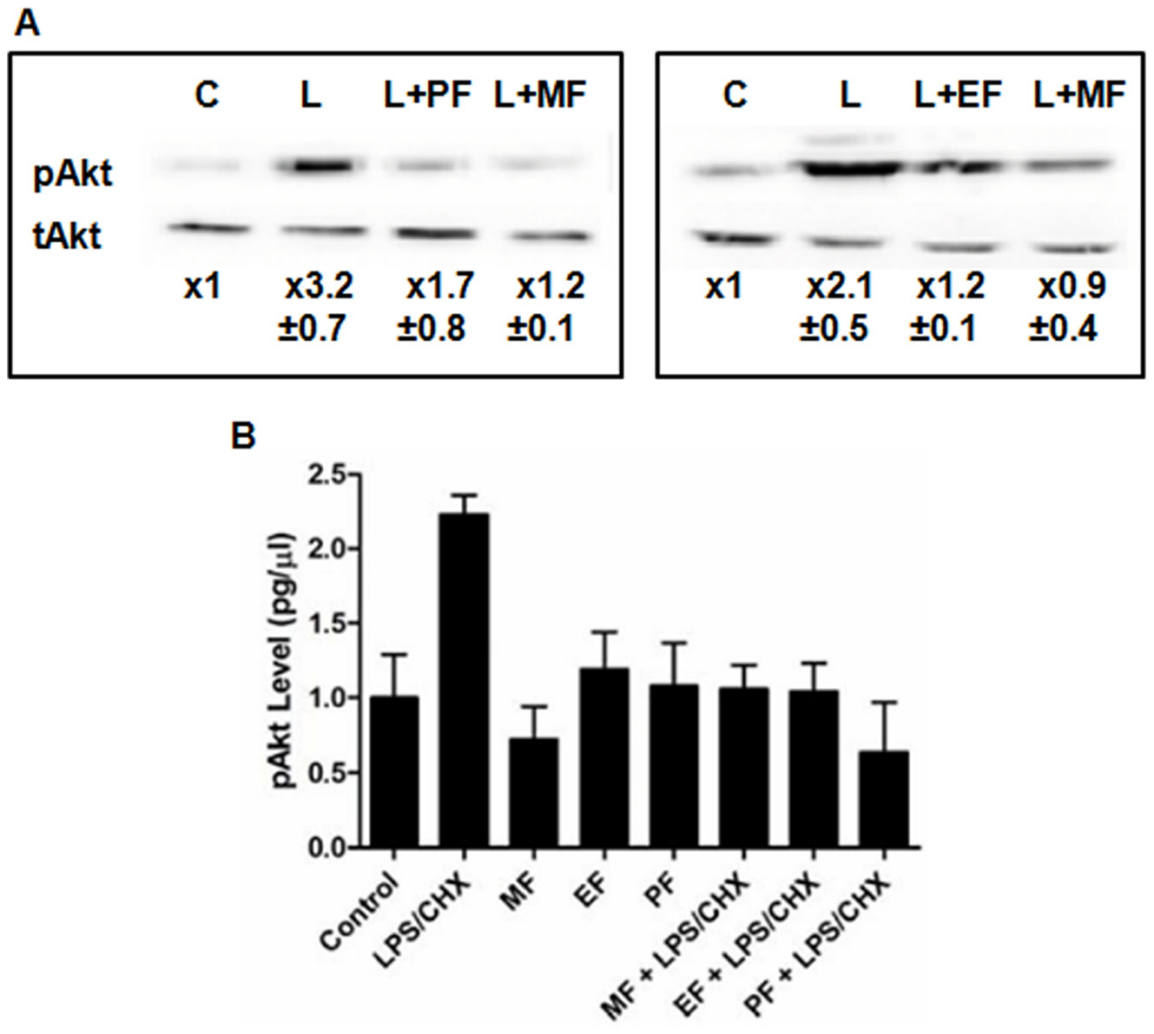
Inhibition of Akt phosphorylation by ALP compounds in HIV-1 vector-transduced or Tat expressing CHME5 cells. (**A**) CHME5 cells transduced with DHIV-GFP vector (D3) giving ∼90% transduction and (**B**) Tat expressing CHME5 subline cells were exposed to LPS (50 µg/ml) and CHX (10 µg/ml) (“L”) in the presence and absence of 25 µM ALP compounds for 24 hours. For the transduced cells (A), the ratios between the levels of phospho-specific and total Akt proteins in the western blots were determined in triplicate, and these ratios were normalized by the ratio in the HIV-1 vector transduced control cells without any treatment (“C”). For the Tat expressing subline cells (B), the phospho-Akt levels were determined by ELISA-based assay, and the phospho-Akt level in the HIV-1 vector transduced control CHME5 cells with no treatment was used for signal normalization. L: LPS and CHX stress treatment, PF: perifosine, EF: edelfosine, MF: miltefosine.

We previously reported that HIV-1 expression promotes the activation of cellular signals involved in the PI3K/Akt pathway in CHME5 cells (e.g. GSK3β phosphorylation, reference 5: Figure S1) and primary human macrophages [5], which explains their elevated cell survival phenotypes. While ALP compounds, which are potential Akt inhibitors, affect cell survival signals in several cancer cell models [13], [15], [16], [17], it has not been tested if the ALP compounds also affect the cell survival signaling in HIV-1 infected cells. Thus, first, ALP compounds were examined for the ability to inhibit the activation of Akt kinase in CHME5 cells, either transduced by the DHIV-GFP vector or expressing Tat protein. CHME5 cells were transduced by an HIV-1 vector, DHIV-GFP, expressing all HIV-1 proteins except Env and Nef; the *nef* gene was replaced with GFP [5], yielding >90% transduction as determined by fluorescence-activated cell sorting (FACS) for GFP expression. At two days post transduction, the transduced cells were treated with lipopolysaccharide (LPS, 50 µg/ml) and cycloheximide (CHX, 10 µg/ml), which impose stress on the cells, in the presence or absence of three different ALP compounds, edelfosine, perifosine or miltefosine (see Figure 1 for their chemical structures), for 24 hours. Lysates were then prepared and applied to western blots for examination of both total Akt and serine-473 phospho-specific Akt levels. Ratios between phospho-specific Akt and total Akt were determined as marked in Figure 2A. Note that since treatment with LPS/CHX alone induced a significant amount of death of the untransduced/control cells within 24 hours as previously reported [19], the analysis of the untransduced cells in this experiment was omitted.

As shown in Figure 2A, the DHIV-GFP vector-transduced cells exhibit a low level of Akt phosphorylation in the absence of LPS/CHX treatment (“C”), while LPS/CHX treatment (“L”) significantly induced the phosphorylation of Akt in the vector-transduced CHME5 cells. However, upon treatment with both LPS/CHX and ALP compounds, the phosphorylation of Akt in transduced cells decreased, suggesting that the ALP compounds effectively block the LPS/CHX stress-induced Akt phosphorylation in the HIV-1 vector-transduced CHME5 cells.

We previously reported that the expression of Tat is responsible for the cytoprotective effect of HIV-1 expressing macrophages and CHME5 cells [5], [19], which is supported by our previous observation that Tat expression elevates the phosphorylation of Akt and GSK3β in CHME5 (Figure S2). Thus, we tested if the ALP compounds also block the stress-induced Akt phosphorylation of the CHME5 sublines that were previously constructed by stably transfecting with pCDNA3.1-hygro [(−) Tat] or pTat101 [(+) Tat] expressing the 101 amino acid M-tropic YU-2 Tat protein [19]. In this test, an ELISA-based phospho-Akt assay was employed to determine the level of Akt phosphorylation. The lysates of (+) Tat CHME5 subline cells were prepared in the presence of LPS/CHX alone or both LPS/CHX and an ALP compound. Lysates, normalized by protein level, were applied for the ELISA-based phospho-Akt assay. Again, note that the control (−) Tat cells were not analyzed in this experiment because the LPS/CHX treatment very effectively induces the death of the control cells as reported previously [19] and shown later in this study. As shown in Figure 2B, the LPS/CHX treatment significantly elevated the Akt phosphorylation in the (+) Tat subline cells, supporting that stress-induced cell survival activation in the Tat expressing CHME5. However, the treatment with the ALP compounds alone did not affect the Akt phosphorylation. It is also important to note that HIV-1 vector transduction alone does not increase the Akt phosphorylation in the absence of the LPS/CHX (Figure 2). However, as shown in Figure 2B, indeed, the treatment with the ALP compounds reduced the LPS/CHX induced Akt phosphorylation in the Tat expressing cells, suggesting the ALP compounds effectively counteract the stress-induced Akt phosphorylation.

### Effect of ALP compounds on kinase activity of Akt in CHME5 cells

**Figure 3:**
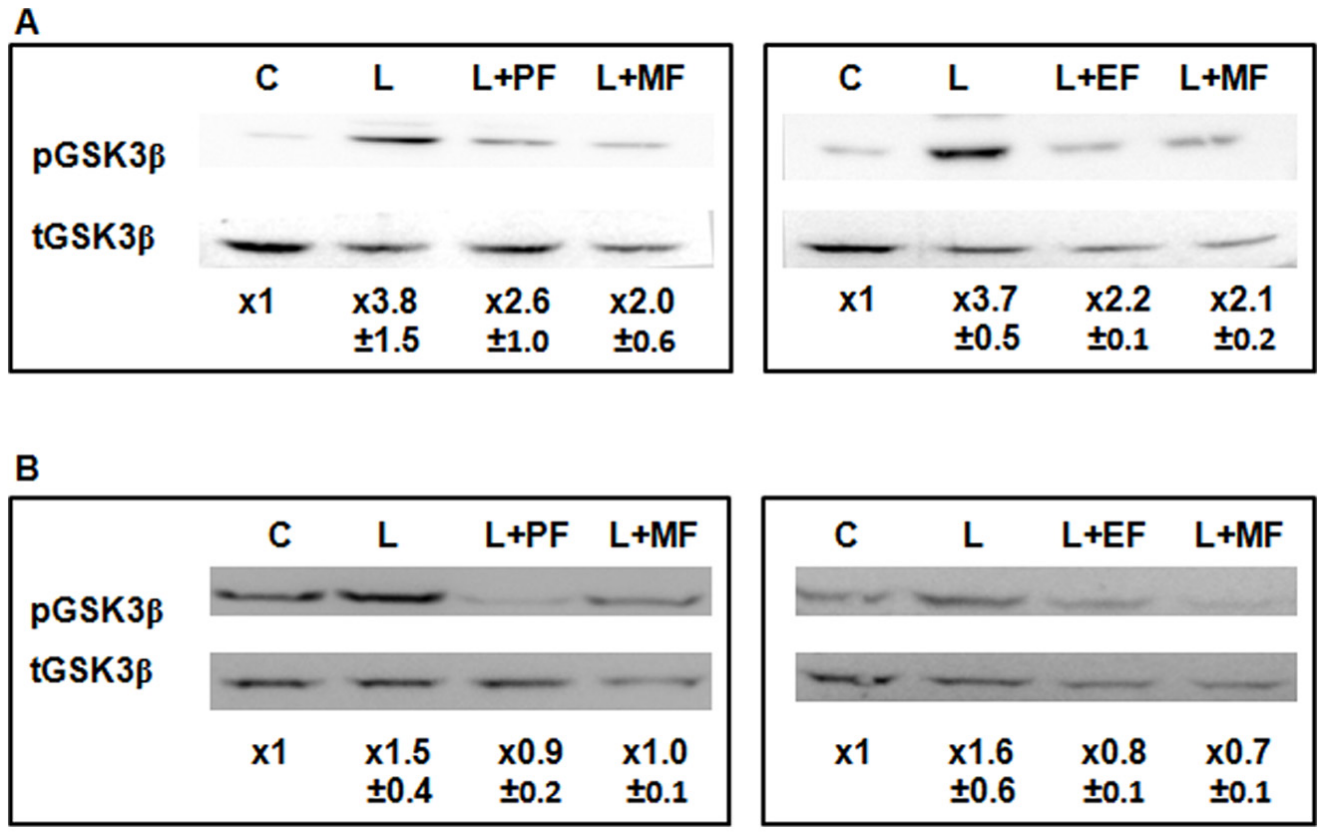
Inhibition of Akt kinase activity by ALP compounds in HIV-1 vector transduced or Tat expressing CHME5 cells. (**A**) CHME5 cells transduced with DHIV-GFP giving ∼90% transduction and (**B**) Tat expressing CHME5 subline cells were exposed to LPS (50 µg/ml)/CHX (10 µg/ml) in the presence or absence of the ALP compounds for 24 hours. Ratios between the levels of phospho-specific and total GSK3β proteins were determined and normalized as described in Figure 2. C: control cells not treated with stress, L: LPS and CHX stress treatment, PF: perifosine, EF: edelfosine, MF: miltefosine.

Phosphorylation of Akt activates its kinase activity, allowing the enzyme to phosphorylate a number of downstream effectors such as GSK3β [20]. We investigated if the ALP compounds, which reduced the phosphorylation of Akt kinase, also block the kinase activity of Akt. For this, the phosphorylation of GSK3β, a downstream substrate of Akt kinase was monitored. The same CHME5 cell lysates used for examining the phosphorylation of Akt kinase (Figure 3) were analyzed for determining both total and phosphorylated GSK3β. As shown in Figure 3A, the LPS/CHX stress elevated the phosphorylation of GSK3β in the DHIV-GFP vector transduced, compared to the untreated cells. However, the treatments with perifosine, edelfosine or miltefosine reduced the phosphorylation of GSK3β even in the presence of LPS/CHX, compared to the LPS/CHX alone treated cells. In addition, as shown in Figure 3B, the three ALP compounds reduced GSK3β phosphorylation to the control cell level in the (+) Tat CHME5 subline cells even in the presence of the LPS/CHX stress. The data shown in Figure 3 support that perifosine, edelfosine, and miltefosine block the kinase activity of Akt in both HIV-1- and Tat-expressing cells. These data mechanistically postulate that the three ALP compounds tested may be able to abolish the cytoprotective phenotype of HIV-1 and Tat-expressing CHME5 cells.

### Counteracting cytoprotective phenotype of CHME5 expressing HIV-1 proteins or Tat by ALP compounds

**Figure 4:**
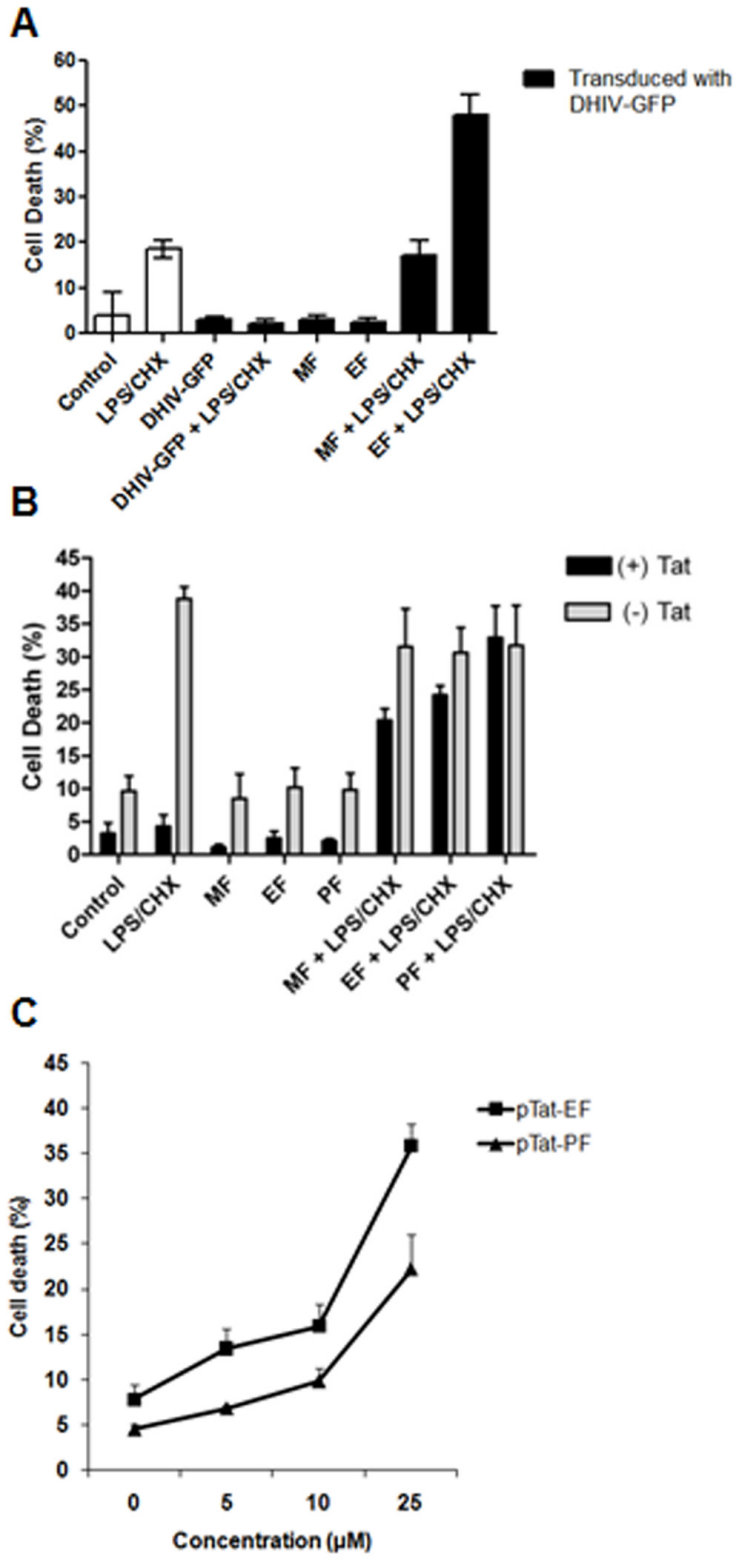
Counteracting effect of ALP compounds against the cytoprotective phenotype of HIV-1 or Tat expressing CHME5 cells. (**A**) Death of CHME5 cells untreated (“Control”) or treated with LPS (50 µg/ml) and CHX (10 µg/ml) (“LPS/CHX”) was determined by staining with ethidium homodimer (red) at 24 hours post treatment. CHME5 cells were also transduced by DHIV-GFP (“D3”, >90% transduction), and treated with LPS/CHX in the presence and absence of 25 µM edelfosine (“EF”) or miltefosine (“MF”) at 48 hours post transduction. The dead cells (red) among the transduced cells (green) were determined to calculate the cell death percentage. (**B**) CHME5 (−) and (+) Tat subline cells were treated with LPS/CHX for 24 hours in the presence or absence of edelfosine (25 µM), perifosine (25 µM) or miltefosine (50 µM). Dead cells were stained with Trypan blue. (**C**) Dose-dependent cell death of the (+) Tat CHME5 subline by edelfosine, perifosine, and miltefosine treatment in the presence of LPS (50 µg/ml) and CHX (10 µg/ml). The percent cell death of the (+) Tat cells treated with both LPS/CHX and different concentrations of the ALP compounds were determined and compared to the percent cell death of the (−) Tat control cells treated with LPS/CHX alone, which gave 39% cell death.

Next, since the data presented in Figures 2 and 3 demonstrated that the ALP compounds inhibit both stress-induced Akt activation and Akt kinase activity, we tested if the ALP compounds counteract the elevated survival capability of the CHME5 cells expressing HIV-1. First, CHME5 cells were transduced by DHIV-GFP (D3) and cultured for 48 hours, yielding >90% transduction as determined by FACS for the GFP expression. Cells were then exposed to LPS and CHX, which impose stress on the cells, for 24 hours in the presence and absence of the ALP compounds (edelfosine and miltefosine, Figure 4A). Cells were stained with ethidium homodimer for visualization of dead cells; the amount of dead cells (red) among transduced cells (green) was determined. As shown in Figure 4A, the LPS/CHX treatment induced cell death (∼20%) in the cells alone, and induced little death among transduced cells (∼2%), supporting the cytoprotective effect of HIV-1 infection in CHME5 cells. Importantly, edelfosine and miltefosine alone did not significantly induce cell death in the transduced cells cultured in the absence of LPS/CHX, suggesting that these compounds do not induce nonspecific cytotoxicity, at least at the concentrations used in Figure 4. However, upon treatment with both edelfosine or miltefosine and LPS/CHX, a high proportion of the transduced cells underwent cell death, suggesting that the ALP compounds effectively abolished the cytoprotective effect of HIV-1 transduction in CHME5 cells.

Next, we tested if the ALP compounds also block the Tat-induced cytoprotective phenotype of CHME5 cells, which was previously reported [5]. In this experiment, cell death of these two sublines was monitored after treatment of perifosine, edelfosine, and miltefosine in the presence and absence of LPS/CHX with a Trypan blue exclusion assay. As shown in Figure 4B, the (−) Tat control CHME5 cells showed a significant cell death (∼40%) by the LPS/CHX treatment, whereas the (+) Tat CHME5 cells displayed a strong protection (<5% cell death) against the LPS/CHX insults, supporting the cytoprotective effect of Tat in CHME5 cells. When treated with perifosine, edelfosine, or miltefosine alone (Figure 4B), both subline cells displayed a background level of cell death, suggesting that these three ALP compounds do not induce nonspecific cytotoxicity in this cell line system at the concentrations used in this experiment. However, upon treatment with both LPS/CHX and the ALP compounds (Figure 4B), the (+) Tat cells lost the Tat-induced cytoprotective effect against the LPS/CHX insult and became sensitive to the LPS/CHX treatment. These data support that all three ALP compounds were able to block the cytoprotective function of Tat in CHME5 cells. Interestingly, the (−) Tat control subline treated with both ALP compounds and LPS/CHX still showed the same high cell death as observed with the treatment of LPS/CHX alone, and thus this indicates that, in contrast to the (+) Tat cells, the ALP compounds do not sensitize the (−) Tat cells to the LPS/CHX insult.

Next, ALP compound-induced sensitization of the (+) Tat CHME5 subline to LPS/CHX was tested for concentration dependent effects. The amount of cell death of (+) Tat cells was determined at 0, 5, 15 and 25 µM of perifosine or edelfosine in the presence of the fixed amount of the LPS/CHX stress. As shown in Figure 4C, the amount of death of the (+) Tat subline cells gradually increases when the concentrations of perifosine and edelfosine increase, and at 25 µM, the ALP compounds re-sensitize the Tat (+) CHME5 cells to the LPS/CHX. Thus, the data shown in Figure 4 support that the ALP compounds induce the loss of the cytoprotective phenotype of both HIV-1 transduced and Tat expressing CHME5 cells.

### Induction of death in HIV-1 infected primary human macrophages by perifosine

Next, we examined if perifosine can also counteract the cytoprotective phenotype of primary human macrophages infected with HIV-1. Importantly, we previously reported that indeed, HIV-1 infected macrophages display activation of Akt (GSK3β phosphorylation and Akt migration to the membrane), and miltefosine was able to effectively counteract the pro-survival phenotype of the HIV-1 infected macrophages [5]. In addition, in this investigation, we chose perifosine (PF) for its clinical significance; PF has been tested through Phase 2 trials for its anti-cancer effect [21].

**Figure 5:**
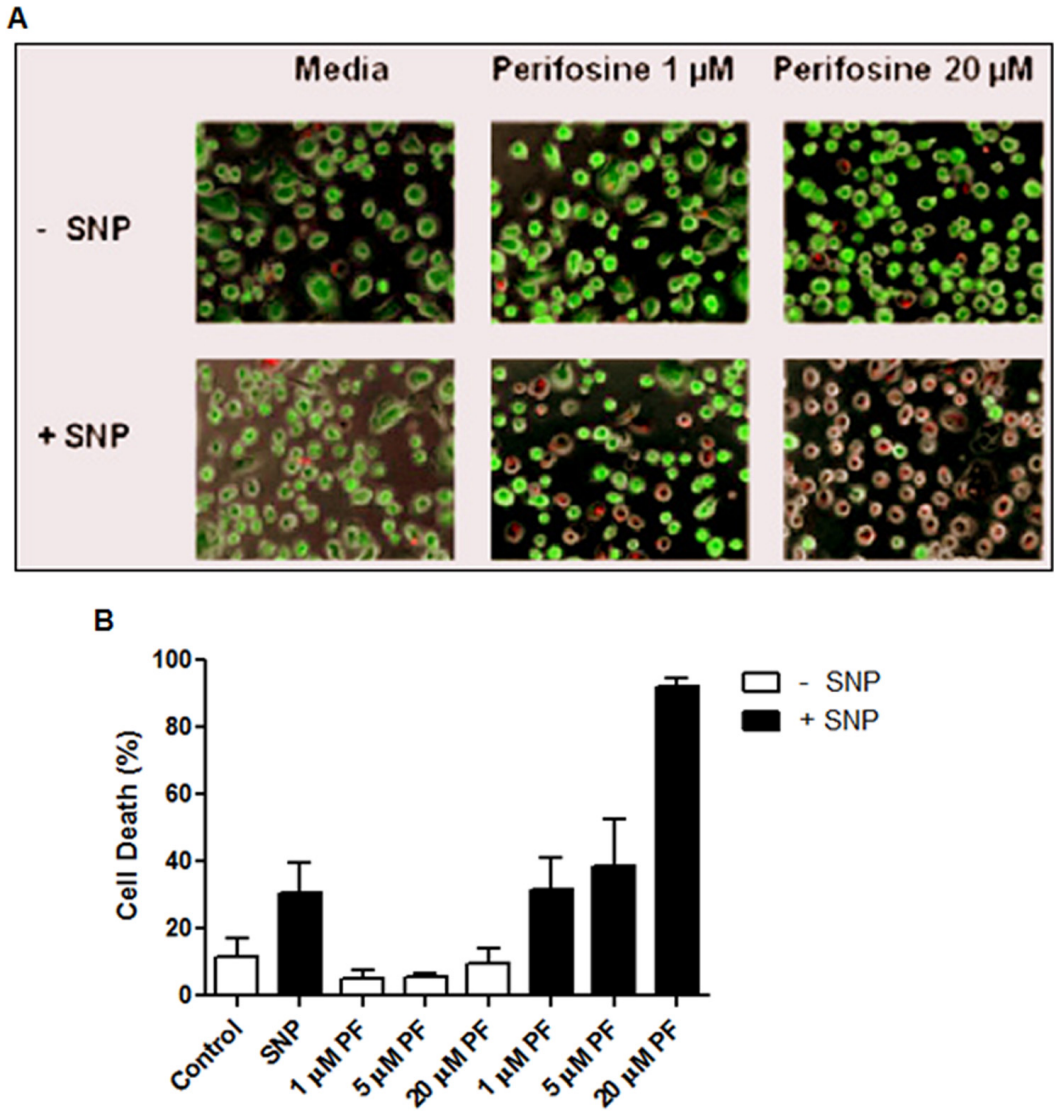
Anti-cytoprotective activity of perifosine in HIV-1 infected primary human macrophages. Primary human monocyte-derived macrophages isolated from blood donors were infected with HIV-1 Bal at MOI 5, and cultured for 12 days in the presence and absence of sodium nitroprusside (SNP, 100 µM) and/or several concentrations of perifosine. Note that 100 µM SNP induces nearly 95% death of uninfected human macrophages [5]. (**A**) Cells were stained with ethidium homodimer and calcein to distinguish between dead (red) and live (green) cells, respectively, on day 12. Images were taken with a fluorescence microscope, and the images shown (merged bright, red, and green fields) are representatives of 3 experiments conducted in duplicate. (**B**) Percent cell death was determined by counting dead and live cells. The values are averages of the duplicates for all conditions performed with macrophages isolated from three separate healthy blood donors. C: infection with no treatment, PF: perifosine.

Primary human monocyte-derived macrophages were infected with 7×10^4^ pg p24 of M-tropic HIV-1 BaL, which is MOI >5 as previously reported [5], and the infected macrophages were cultured for 12 days in various conditions shown in Figure 5. Under these culture conditions, more than 95% of the macrophages were infected at 12 days post infection as determined by the p24 intracellular staining (data not shown). The cells were then analyzed for live and dead phenotypes by staining with live (green) and dead (red) cell specific dyes. In this study, three concentrations of perifosine were used, 1, 5 and 20 µM, and 100 µM sodium nitroprusside (SNP) which is a nitric oxide producer, well known to be an HIV-1 related cellular stress [22].

As shown in Figure 5A and 5B, HIV-1 infected macrophages showed effective cell survival (green cells) for 12 days in the absence of SNP and perifosine (media alone), which is contrasted to the HIV-1 infected CD4^+^ T cells known to undergo cell death within a few days post infection. Under this culture condition, the basal level of death of HIV-1 infected macrophages was ∼10%. Furthermore, HIV-1 infected macrophages displayed strong survival (green) with only a slight increase (<2-fold) of the basal cell death (Figure 5A and 5B) even in the presence of the 100 µM SNP stress which normally induces >90% cell death in the uninfected macrophages [19], confirming the cytoprotective phenotype of the HIV-1 infected macrophages. When the infected macrophages were treated with the three different concentrations of perifosine without SNP, the cells still exhibited only background cell death (Figure 5A and 5B), suggesting that perifosine does not impose nonspecific cytotoxicity on the infected macrophages at these concentrations. However, simultaneous treatment of perifosine and SNP to the culture revealed elevated death compared to the perifosine or SNP singly treated control cells (Figure 5A). Moreover, cell death increased in a dose-dependent manner with a rise in perifosine concentration in the presence of SNP (Figure 5B). Thus, the data in Figure 5 suggest that perifosine induces the death of HIV-1 infected macrophages, specifically when the cells were exposed to cellular stress which does not normally induce the death of the HIV-1 infected macrophages.

### Effect of ALPs on HIV-1 production in macrophages

**Figure 6:**
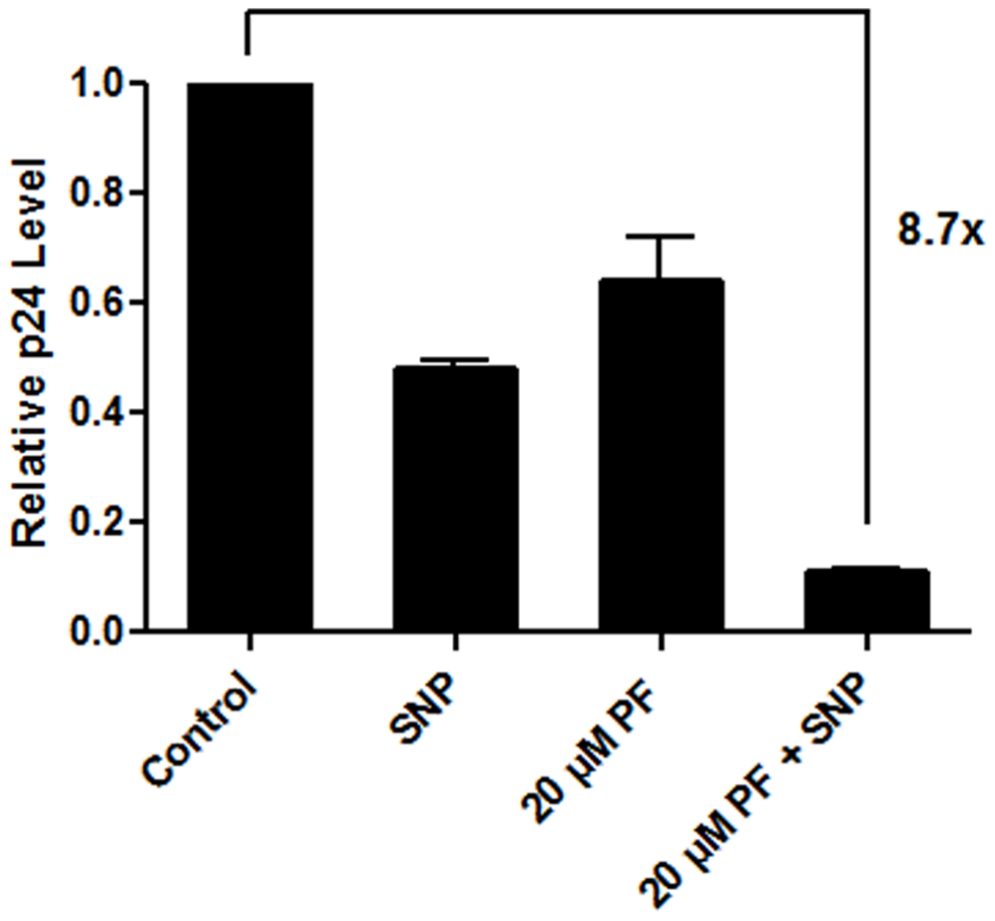
Inhibition of HIV-1 production from primary human monocyte-derived macrophages by perifosine. Primary human monocyte-derived macrophages were infected and cultured for 12 days in the presence and absence of sodium nitroprusside (SNP, 100 µM) and/or perifosine (20 µM), and the p24 levels of the supernatants on the 12^th^ day of the culture were determined. The p24 level at day 12 for each condition was normalized by the p24 level (86 pg/ml) of the media only control (no treatment).

The data presented in Figure 5 demonstrate that perifosine induces death of HIV-1 infected macrophages when they are exposed to a level of cellular stress which alone does not kill the HIV-1 infected macrophages. Thus, finally, we tested if perifosine is able to reduce viral production from HIV-1 infected macrophages in the presence of SNP stress. Primary macrophages were infected with a high MOI of M-tropic HIV-1BaL, and monitored viral production by a p24 ELISA assay after 12 days of culture in the presence or absence of perifosine (20 µM) and SNP (100 µM). As shown in Figure 6, SNP treatment reduced viral production only by ∼50%, which is consistent with the previous report that the SNP treatment usually decreases viral production 50–70% while the cell death assay showed <5% death of the HIV infected macrophages (Figure 5) [5]. Additionally, 20 µM perifosine alone only slightly affected viral production. However, when HIV-1 infected macrophages were exposed to both perifosine and SNP, a 9-fold reduction of viral production was observed, which should be a consequence of the significant cell death in the HIV-1 infected macrophages (Figure 5). Therefore, these data observed with primary human macrophages and infectious HIV-1 support the idea that perifosine effectively reduces HIV-1 production by antagonizing the stress-induced cytoprotective phenotype of the HIV-1 infected macrophages.

## Discussion

Targeting host factors that are utilized for the life cycle of HIV-1 is a highly attractive anti-viral strategy. Unlike current anti-viral treatments targeting viral proteins, such as reverse transcriptase, protease and integrase, this strategy can minimize viral escape. One major obstacle in targeting host factors is that many which are utilized by HIV-1 are also essential to the host. Thus the conditionally required or non-essential host factors are better candidates as anti-HIV-1 targets. Indeed, the Akt cell survival pathway is conditionally activated only upon exposure to cytotoxic insults or in abnormal cells that require exceptional survival techniques, such as cancer cells.

Importantly, it is well known that HIV-1 infected macrophages and microglia establish toxic extracellular environments by persistently secreting viral cytotoxic proteins (e.g. gp120 and Tat) and chemicals (e.g. NO), and this toxic environment induces death of nearby neurons in the CNS, leading to HIV-1 associated neurodegenerative diseases [2]. Ironically, even with the establishment of extracellular cytotoxic conditions HIV-1 infected macrophages exhibit an extended life span, (unlike infected CD4^+^ T cells which undergo cell death) and serve as long-term HIV-1 reservoirs [1], [19], [23]. Thus it is highly plausible that the pro-activation capability of the stress-induced PI3K/Akt cell survival pathway by HIV-1 infection can effectively protect the infected macrophages from the toxic local environments generated by their own proteins and chemicals, leading to long-lived viral reservoirs.

In this report, we demonstrated that the ALP compounds, edelfosine, perifosine and miltefosine, were able to inhibit HIV-1 induced Akt activation, leading to the death of HIV-1 expressing macrophages and CHME5 cells. However, edelfosine treatment of primary human macrophages proved to be significantly cytotoxic at concentrations as low as 1 µM (data not shown). The precise mechanism of action of ALP compounds against Akt kinase remains unclear, though multiple reports demonstrated that the compounds reduce Akt activation in tumor tissue culture models [13], [14], [15], [16]. While our study focused on the inhibitory function of ALP compounds against activation of the Akt cell survival pathway, we do not exclude a possibility that the inhibitory effect of these two ALP compounds on the cytoprotective phenotype of HIV-1 expressing macrophages and CHME5 cells may also result from other closely related cellular events such as the activation of the apoptotic pathway and inhibition of the anti-apoptotic pathway. In fact, whether HIV-1 infection mechanistically affects apoptosis-related events in macrophages has not been tested, presumably because unlike CD4^+^ T cells undergoing cell death following G2 arrest, the cell death phenotype of HIV-1 infected macrophages is absent (these cells actually live longer). Since we employed ethidium homodimer which primarily stains necrotic cells, not living or apoptotic cells, our study monitored the necrosis rate of the HIV-1 infected macrophages upon exposure to the ALP compounds. Indeed, the effect of HIV-1 infection as well as the ALP treatment on apoptosis and anti-apoptosis can be investigated in the future. In addition, while our data demonstrate that the ALP compounds effectively inhibit the HIV-1 and Tat-induced Akt activation/phosphorylation in the CHME5 model, it still remains to be confirmed that the ALP compounds also block Akt phosphorylation in HIV-1 infected macrophages. This is mainly due to technical issues: we found that the primary human macrophage lysates, particularly, after ALP treatment, were not applicable to various methods of Akt activity including western analysis.

The ALP compounds investigated in this study are known to be well-tolerated in various pre-clinical trials [24] and to do little harm in normal cells in various in vitro models; this could be due to the fact that normal cells do not activate the PI3K/Akt pathway unless they are exposed to extracellular insults. Importantly, many studies, especially from anti-Leishmania research, demonstrated that ALP compounds are effectively able to penetrate the blood-brain barrier (BBB) [25], [26]. Considering that HIV-1 infected macrophages and microglia in the brain play a key role in HAND, this BBB penetration feature of ALP compounds is very important in terms of their potential clinical application. However, it is clear that further investigations and evaluations of the ALP compounds as anti-HIV agents need to be conducted.

## Supporting Information

Figure S1 Akt and GSKb phosphorylation by DHIV-GFP in CHME5cells. 1×105 CHME5 cells were transduced by DHIV-GFP as described in Figure 2, and the cell lysates were analyzed by western blots for total (t) and phospho (p) specific Akt and GSK3β. The fold differences in the signals are marked.
(1.56 MB TIF)

Figure S2 Akt and GSKβ phosphorylation by Tat expression in CHME5 cells. Lysates from 1×105 CHME5 sublines with pCDNA3.1-hygro (-) or pTat101 (+) cells were analyzed by western blot for total (t) and phospho (p) specific Akt and GSK3β, as described in Figure 3. The fold differences in the signals are marked.
(1.56 MB TIF)
