# Supplementary figures and images for: Targeting the PI3K/Akt Cell Survival Pathway to Induce Cell Death of HIV-1 Infected Macrophages with Alkylphospholipid Compounds
Source: PLoS One. 2010 Sep 30;5(9):e13121. doi: 10.1371/journal.pone.0013121 (PMC2948033; doi:10.1371/journal.pone.0013121)

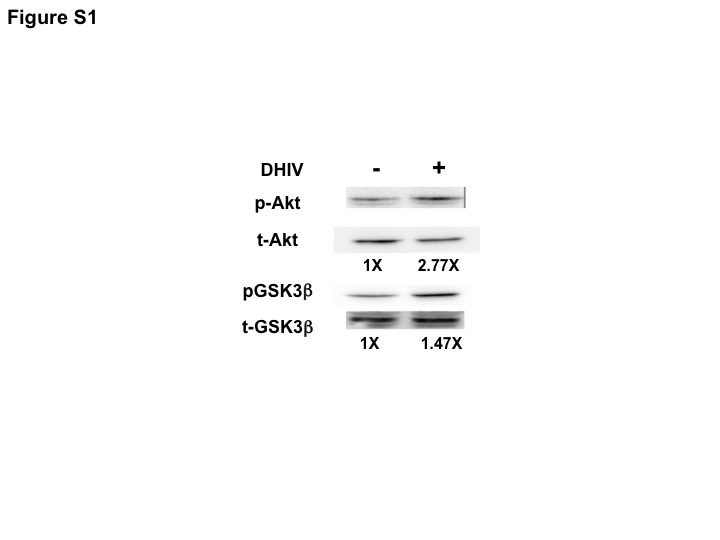

Supplement: Figure S1 — Akt and GSKb phosphorylation by DHIV-GFP in CHME5cells. 1×10^5^ CHME5 cells were transduced by DHIV-GFP as described in Figure 2, and the cell lysates were analyzed by western blots for total (t) and phospho (p) specific Akt and GSK3β. The fold differences in the signals are marked. (1.56 MB TIF) [file pone.0013121.s001.tif]

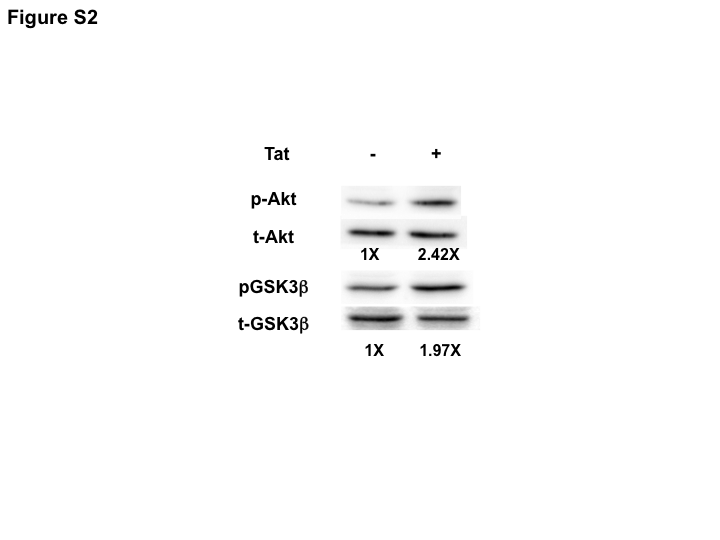

Supplement: Figure S2 — Akt and GSKβ phosphorylation by Tat expression in CHME5 cells. Lysates from 1×10^5^ CHME5 sublines with pCDNA3.1-hygro (-) or pTat101 (+) cells were analyzed by western blot for total (t) and phospho (p) specific Akt and GSK3β, as described in Figure 3. The fold differences in the signals are marked. (1.56 MB TIF) [file pone.0013121.s002.tif]
